# Synergies between optical and physical variables in intercepting parabolic targets

**DOI:** 10.3389/fnbeh.2013.00046

**Published:** 2013-05-16

**Authors:** José Gómez, Joan López-Moliner

**Affiliations:** ^1^Departament de Matemàtica Aplicada IV, Universitat Politècnica de CatalunyaBarcelona, Spain; ^2^Institute for Brain, Cognition and Behaviour (IR3C) and Departament de Psicologia Bàsica, Universitat de BarcelonaBarcelona, Spain

**Keywords:** interceptive timing, parabolic trajectories, prior knowledge, time-to-contact, 3D motion perception

## Abstract

Interception requires precise estimation of time-to-contact (*TTC*) information. A long-standing view posits that all relevant information for extracting *TTC* is available in the angular variables, which result from the projection of distal objects onto the retina. The different timing models rooted in this tradition have consequently relied on combining visual angle and its rate of expansion in different ways with tau being the most well-known solution for *TTC*. The generalization of these models to timing parabolic trajectories is not straightforward. For example, these different combinations rely on isotropic expansion and usually assume first-order information only, neglecting acceleration. As a consequence no optical formulations have been put forward so far to specify *TTC* of parabolic targets with enough accuracy. It is only recently that context-dependent physical variables have been shown to play an important role in *TTC* estimation. Known physical size and gravity can adequately explain observed data of linear and free-falling trajectories, respectively. Yet, a full timing model for specifying parabolic *TTC* has remained elusive. We here derive two formulations that specify *TTC* for parabolic ball trajectories. The first specification extends previous models in which known size is combined with thresholding visual angle or its rate of expansion to the case of fly balls. To efficiently use this model, observers need to recover the 3D radial velocity component of the trajectory which conveys the isotropic expansion. The second one uses knowledge of size and gravity combined with ball visual angle and elevation angle. Taking into account the noise due to sensory measurements, we simulate the expected performance of these models in terms of accuracy and precision. While the model that combines expansion information and size knowledge is more efficient during the late trajectory, the second one is shown to be efficient along all the flight.

## Introduction

The time remaining before a moving target arrives at some point of interest is known as the time-to-contact (*TTC*) with that place. An accurate estimate of this quantity could be used to control an interceptive or evasive action directed at that target. How people and animals perceive *TTC* has been the primary motivation of a large number of studies mainly inspired by the Ecological Psychology framework (e.g., Lee, 1976; Gibson, 1979; Bootsma and Oudejans, 1993; Peper et al., 1994). Nevertheless, the adopted stance to tackle this general problem has often neglected deliberately to recover 3D information and has rather focused on extracting invariants from optical or angular variables. The most successful model within this tradition has been the τ model (Lee, 1976) which specifies that *TTC* can be estimated as the ratio between visual angle θ and its rate of expansion $\dot{\theta }$. Despite its known restrictions (e.g., it neglects acceleration and fails for large visual angles) τ has been essential to *TTC* research for a long time. At the neurophysiological level, neurons that respond to *TTC* in a τ-like way (i.e., independently of size and velocity) have been also reported (Sun and Frost, 1998) and recently τ has even inspired the development of more elaborated and biologically plausible computational implementations of *TTC* estimation (Keil and López-Moliner, 2012). Behaviorally speaking, however, a bulk of recent evidence casts doubts on the use of τ as a general model for *TTC* estimation. Deviations from τ predictions have been reported depending on the type of task (Lugtigheid and Welchman, 2011), the duration of the trajectory (Hosking and Crassini, 2011), illusory or contextual effects (Smeets et al., 1996; DeLucia et al., 2000), texture (Jacobs and Diaz, 2010) or reliability of physical information relevant for catching like ball size (López-Moliner et al., 2007b; López-Moliner and Keil, 2012) or shape (López-Moliner et al., 2007a).

Accumulation of evidence against τ has, partly, been stimulated by the necessity of recovering three-dimensional (3D) contextual information. For example, Wann (1996) already proposed an alternative to τ based on the use of traveled physical distance. The strategy that he proposed gained further validity in López-Moliner and Keil (2012), where the action of catching was initiated at a constant distance when physical size was known. It seems then that some assumptions about 3D information, physical size in this case, make subjects switch to using simpler computations for *TTC* estimation without the need to combine different angular variables. Interception often implies the interaction with solid objects moving in the 3D external space. Not only do we then need to update the position in the 3D space continuously but also require 3D knowledge of the object to be caught (e.g., shape and size) so as to control our hand movements. It is only recently that 3D information is regarded as being relevant in the context of *TTC* estimation, once attention has been somehow shifted from pure *TTC* perception to interceptive control mechanisms in interception studies (Tresilian, 2004).

The recovery of 3D structure or 3D motion from the optical 2D ambiguous images generated by objects on the retina is a long-standing problem that is the essence of the constructivist inferential approach to perception. This probably explains the minor role of 3D information in *TTC* studies. The process of 3D recovery is complex and sometimes is misleadingly illustrated by the potentially infinite solutions consistent with a single retinal image. The ecological theory, however, circumvents this problem and instead exploits useful relations and constraints that are present in the optic array, but at the cost of being unable to propose valid *TTC* specifications for parabolic trajectories. Parabolic balls are ubiquitous in ball games and in many daily-life situations as well. Nevertheless, an accurate specification of *TTC* for fly balls has been missing thus far.

In this paper we put forward two *TTC* specifications and show that one of them is viable as a general model for catching fly balls in terms of accuracy and precision. Both models make use of context-specific information in addition to optical information. One extends previous models which make use of known size to compute *TTC* (López-Moliner et al., 2007b; López-Moliner and Keil, 2012) to fly balls. This model relies on knowing physical size to recover 3D speed components of the parabolic trajectory. After showing that this first model is unable to deal with high parabolae or provide useful predictive information in case of early occlusion, we introduce a second model that relies on physical size and gravity. A hallmark of this model is that it does not need rate of expansion which makes it very robust to noise, even in the early part of the trajectory. This model, as a consequence, encapsulates reliable predictive information allowing the use of predictive and online control mechanism in interception.

### Timing parabolic balls from physical information

**Figure 1:**
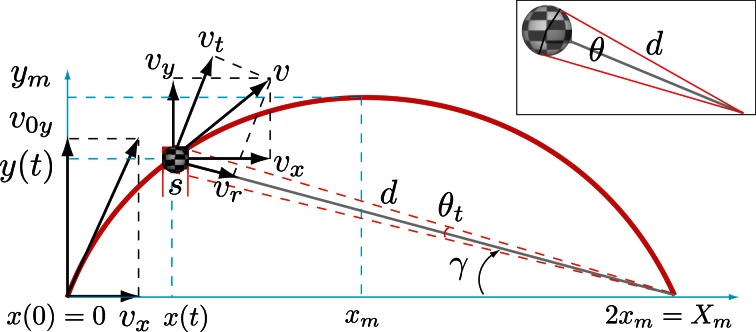
**An example of a parabolic path of a ball of size *s* with initial vertical velocity *v*_0*y*_ and horizontal velocity *v*_*x*_.** *v* is the tangential velocity with respect to the trajectory, *v*_*r*_ is the radial component and *v*_*t*_ is tangential velocity component with respect to the point of observation (normal to *v*_*r*_). The path reaches its maximum height *y*_*m*_ when it has covered an horizontal distance of *x*_*m*_ and will return to its initial height after covering an horizontal distance of 2 *x*_*m*_. The picture shows the different velocity components for an elevation angle γ at a distance *d* to the point of observation at which time the ball subtends an angle θ. (inset) Illustration of the relation between size *s*, the angle θ and distance *d* as defined in Appendix A (Equation A.1).

Unlike the case of linear trajectories, the specification of visual information that is useful for timing a parabolic catch has been much less studied. Nevertheless parabolic trajectories have been objects of attention in several studies that have examined how catching performance depends on vision of different parts of the parabolic path (e.g., Sharp and Whiting, 1974, 1975; Whiting and Sharp, 1974; Dessing et al., 2009; López-Moliner et al., 2010) or which visual information in fly balls is used to predict the landing point (Chapman, 1968; Oudejans et al., 1997; McLeod et al., 2003; Brouwer et al., 2006; Fink et al., 2009). However, putting forward a computational model that specifies how humans can time the parabolic catch has remained elusive. One possible reason could be that we can only derive temporal measures for fly balls by using physical information that is neither optically nor perceptually available. Figure 1 shows an example of a parabolic path and its decomposition in different velocity vectors. Certainly, if we ignore air resistance (we address this point later on) and assuming that the ball starts moving at eye height, the time that the ball remains above eye height is:

(1) $$\begin{array}{l} T=\frac{2v_{0y}}{g} \end{array}$$

where *v*_0*y*_ is the initial vertical velocity (see Figure 1) and *g* is the acceleration caused by gravity. However, and despite having an expression that physically determines a time quantity that could provide an approximation of initial *TTC*, the use of Equation (1) to time a catch encounters different problems. The main one possibly is the need for an accurate estimate of *v*_0*y*_, that is the velocity at movement onset assuming the fly starts around eye height. Estimating the initial vertical velocity is difficult because of two problems. First, unlike the horizontal velocity (*v*_*x*_), the vertical velocity is not constant but rather decelerates first and accelerates after reaching the maximum height while it is the initial value which is the relevant one. The perception of velocity changes takes some integration time (Werkhoven et al., 1992) which could compromise successful catching when time is in short supply. Secondly, there is the problem of how to estimate *v*_0*y*_. One possibility is to use angular correlates like the rate of change of the elevation angle $\dot{\gamma }$ scaled with distance (e.g., Brouwer et al., 2006; Zago et al., 2009), however, it is not clear how the error from a depth estimate would affect *TTC* through the vertical velocity. We comment on this point again later when introducing the model that incorporates gravity.

Therefore it is not clear how people can estimate the initial value of the vertical velocity accurately enough. In the following we outline the two potential mechanisms that could be used to estimate *TTC* in parabolic trajectories. The first one is an extension of previous models that combine prior knowledge of size and optical thresholds. In this case, distance is not necessary and 3D motion can be estimated from optical variables by assuming size knowledge. After showing that this first model has limited application, we introduce a more general model which includes gravity and show that it is viable for accurate catching.

## Optical thresholds and known size (*KS* model)

It is very likely that, with extensive practice, we quickly acquire information on steady-state attributes of the objects (e.g., physical size) that we have to catch. Within a given context, the visual system could therefore exploit lawful relations between physical and optical variables. For example, for linearly approaching objects moving at constant velocity, one such a law (López-Moliner et al., 2007b) that holds between size (*s*), physical velocity (*v*) and the resulting optical variables (θ and $\dot{\theta }$) is:

(2) $$v=\frac{s\dot{\theta }}{\theta^{2}}$$

Although Equation (2) does not directly signal *TTC*, the accurate time can, in principle, be estimated once velocity is recovered for a known size object in two different ways:

(3) $$TTC\approx \frac{1}{\theta_{th}}\frac{s}{v}$$

or

(4) $$\begin{array}{l} TTC\approx \frac{1}{\sqrt{{\dot{\theta }}_{th}}}\sqrt{\frac{s}{v}} \end{array}$$

where θ_*th*_ and ${\dot{\theta }}_{th}$ are visual angle and rate of expansion thresholds. Once these thresholds are reached there is some known time left until contact for a given size and velocity. Therefore, once size and velocity are known, accurate timing can be obtained by thresholding action initiation on the visual angle (θ) or its rate of expansion ($\dot{\theta }$), respectively for Equations (3) and (4). It has been recently shown (López-Moliner and Keil, 2012) that both expressions define a continuum in which subjects can dynamically put more weight on either optical variable depending on the temporal constraints of the task once physical size is known. In this sense, when there is little time for the action to unfold (very short *TTC*) people favor $\dot{\theta }$ to reduce the temporal error at the time of initiation, otherwise a θ threshold (θ_*th*_) is used to initiate the movement. However, the use of Equations (3) and (4) depends on people recovering physical velocity which is even less straightforward for parabolic trajectories. In addition, we have different velocity components along the movement of a fly ball (i.e., tangential, vertical, horizontal, and radial; see Figure 1) with the horizontal velocity (*v*_*x*_) being the only component that remains constant along the flight. Although approximations of both the vertical (initial) and horizontal velocities have been previously proposed (Brouwer et al., 2006) to solve the outfielder problem (i.e., to differentiate between balls landing behind and those landing in front of the catchers), neither component by itself generates an isotropic expansion to allow people to use Equations (3) and (4) after accurately recovering velocity. The radial velocity (*v*_*r*_) component (see Figure 1), albeit unperceivable, does indeed carry the isotropic expansion of the projected image on the retina. If *v*_*r*_ can be estimated then we could countenance the use of previous models to some parabolic cases.

Just as it is known that thresholding θ and $\dot{\theta }$ can be encapsulated in a unified expression for linear trajectories (López-Moliner and Keil, 2012), here we show so for the parabolic case (see Appendix A for a full mathematical derivation). As a result, it can be shown that the following expression signals *TTC* and includes the strategies based on θ and $\dot{\theta }$ thresholds introduced above:

(5) $$\begin{array}{l} TTC=\frac{s}{v_{r}\theta +s\dot{\gamma }\tan\gamma } \end{array}$$

As can be noted Equation (5) includes physical terms (i.e., ball size *s* and radial velocity *v*_*r*_) as well as optical terms (i.e., the elevation angle γ, its time derivative, $\dot{\gamma }$ and, visual angle θ). Note that it is very straightforward to show that when the rate of change of the elevation angle $\dot{\gamma }$ is very small or zero (i.e., horizontal motion) Equation (5) becomes Equation (3). In Appendix A we show that Equation (5) also includes Equation (4). Leaving aside how to estimate *v*_*r*_, the estimation of *TTC* from Equation (5) does not depend on the rate of change of the visual angle which is the noisiest optical measurement (discrimination thresholds of about 10%) especially when the visual angle is very small and does depend on angular or optical measurements that are less noisy. For example, Weber fractions of about 5% have been reported for judgments of $\dot{\gamma }$ for values between 0.03 and 1.2 rad/s (McKee, 1981) and even smaller for judgments of the visual angle θ (McKee and Welch, 1992). In the same vein, humans can detect values of $\dot{\gamma }$ as small as 0.0003 rad/s (McKee, 1981; Regan, 1997). As to optical measurements, therefore, these error values render the model described in equation 5 worthy of further testing.

**Figure 2:**
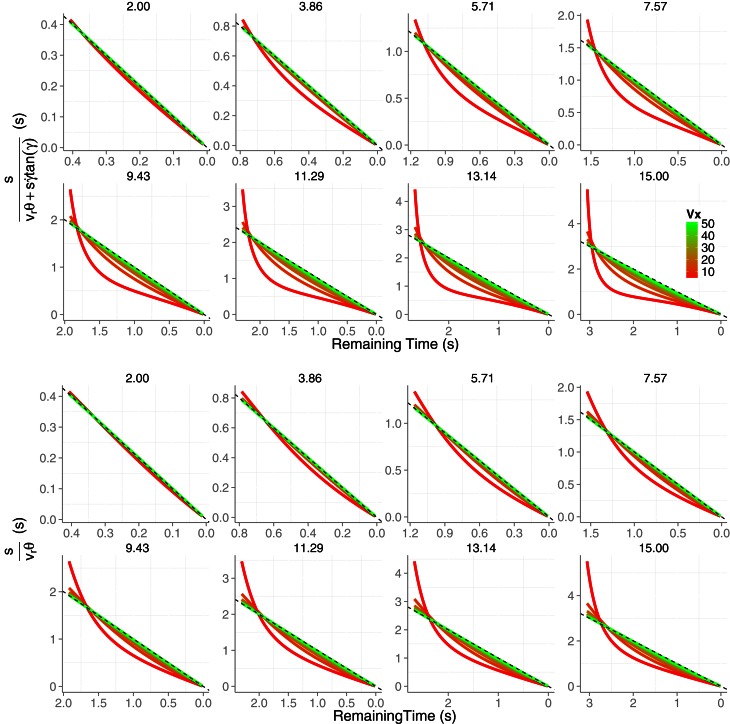
**Time-to-contact estimates across time obtained from the model described in Equation (5) (two top rows).** Different panels correspond to the different eight initial vertical velocities *v*_0*y*_. In each panel the predictions for the different eight horizontal velocities *v*_*x*_ are color-coded. In the two bottom rows we plot the same *TTC* estimates obtained from simplifying Equation (5) by nullifying $\dot{\gamma }$ which renders Equation (5) equivalent to Equation (3). The dashed (unity) line denotes the remaining actual TTC. Axes have different scales from one panel to another to increase readability.

In Figure 2 (top panels) we show the *TTC* predictions derived from Equation (5). The predictions correspond to parabolic trajectories of a tennis ball (6.6 cm of diameter) that resulted from combining eight initial values of vertical velocities *v*_0*y*_ (2, 3.86, 5.71, 7.57, 9.43, 11.29, 13.14, and 15 m/s) with eight horizontal velocities *v*_*x*_ (5, 11.43, 17.86, 24.29 30.7, 37.14, 43.57, and 50 m/s). These values were chosen from simulated trajectories that are likely to happen in a tennis game. The trajectories are simulated to start at eye height and the initial *TTC* is set to the time that the ball remains above the initial height (i.e., contact at observer's eyes). As can be seen, expression 5 signals *TTC* accurately especially for high horizontal velocities or when the initial vertical velocity is small. For example, for *v*_0*y*_ = 2 m/s the prediction of the remaining *TTC* is very accurate for all horizontals velocities (all lines are very close to the dashed line which denotes the remaining time above eye height). Therefore, Equation (5) in principle signals *TTC* accurately if *v*_*x*_ >> *v*_0*y*_, e.g., a football ball that is kicked at a shallow angle and will not go very high. This is not surprising as Equation (5) generalizes previous models outlined for linear (horizontal) trajectories. Actually, the *TTC* estimations for the simplified version of the model represented by Equation (5), that is, $\dot{\gamma }=0$ are shown in the bottom panels. It can be actually seen that the deviation from the dashed line is less pronounced for the slower horizontal velocities (i.e., higher parabolae). Hereafter we will use the simplified version of Equation (5), that is we will set $\dot{\gamma }=0$ in the *KS* model.

### Estimation of 3D radial velocity from known size

The feasibility of using the *KS* model relies on recovering the radial velocity *v*_*r*_ which conveys the pure isotropic expansion. Whether or not people can perceive accurately the velocity of an object in the 3D environment has been a long-standing matter of debate and there is no clear answer yet. For example, McKee and Welch (1989) reported that judgments of 3D lateral speed were biased toward retinal speed undermining velocity constancy. A review of other studies in Howard and Rogers (2001) shows, however, that improvements of 3D speed judgments are possible with richer stimulation. More recently Rushton and Duke (2009) concluded that subjects cannot judge the 3D speed of approaching objects and reported weber fractions between 0.16 and 0.23 for size-varying objects. While this precision is indeed useless for real catching, it is also the case that size was randomized (up to ±20%) in this study to prevent subjects from using $\dot{\theta }$ as a cue to velocity. However, this large variability of size could have discouraged subjects from exploiting lawful relations (e.g., Equation 2) to estimate 3D speed. It is worth at least exploring, therefore, to what extent people could recover radial speed from the known size and optical variables.

**Figure 3:**
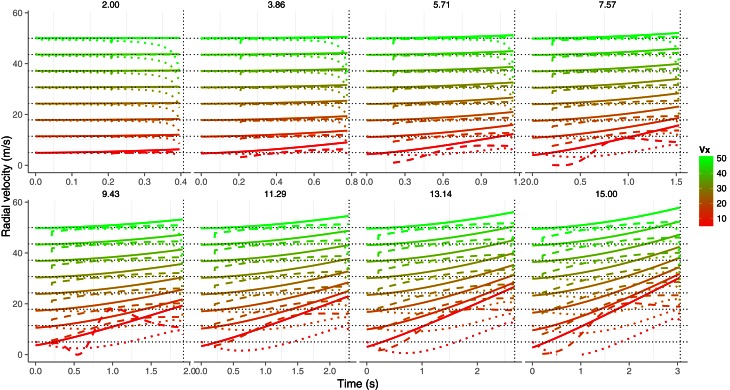
**The time course of radial velocity (solid lines) and its estimation from known size and optical variables by using Equation (2) (color dotted lines) and using Equation (6) (dashed lines).** Different initial vertical velocities *v*_0*y*_ are plotted in separate panels. The eight horizontal dotted black lines denote the different horizontal velocities *v*_*x*_ and, a vertical dotted line is placed in each panel at the *TTC* value at the start of the trajectory which depend on the initial vertical velocity (e.g., the shorter initial *TTC* is 0.407 s and corresponds to a *v*_0*y*_ of 2 m · s^−1^). The estimation from Equation (6) was computed by comparing two samples of $\dot{\theta }$ and then using Equation (6). The time interval (Δ*t* = *t*_2_ − *t*_1_) between the two consecutive $\dot{\theta }$ samples was 96 ms. Color codes different horizontal velocities. See text for details.

Equation (2) specifies one possible way in which velocity can be estimated and this relation could also hold for *v*_*r*_. Figure 3 shows the time course of radial velocity (solid lines) for the same trajectories used previously. It can be seen that, on average, the radial speed grows and deviates from the corresponding horizontal velocities *v*_*x*_ (horizontal black dotted lines) as *v*_0*y*_ gets higher (different panels). Leaving aside for the moment, the effects of noise in optical measures, Equation (2) underestimates the radial velocity (dotted lines in Figure 3) and only provides good predictions for slow values of *v*_0*y*_ (e.g., 2.0 m/s) but even then the estimation falls short as the ball contact approaches. For example, when *v*_0*y*_ is 5.71 (third panel, first row in Figure 3), *v*_*r*_ is underestimated by about 45% 150 ms before contact $\left({\hat{v}}_{r}=6.01,\;v_{r}=10.97\text{ m/s}\right)$ for the slowest horizontal velocity *v*_*x*_ (5 m/s). At the same time before contact, the underestimation is about 8% for the fastest horizontal speed (50 m/s). Taken all together, *v*_*r*_ can only be recovered accurately using Equation (2) in very shallow trajectories.

Alternatively, López-Moliner et al. (2007b) showed that 3D approaching velocity can also be obtained in linear trajectories by comparing successive samples of $\dot{\theta }$ within a time window Δ*t*:

(6) $$v=\frac{s{\left(\sqrt{\frac{1}{{\dot{\theta }}_{1}}}-\sqrt{\frac{1}{{\dot{\theta }}_{2}}}\right)}^{2}}{\Delta t^{2}}$$

In practical terms Equation (6) implies ascertaining a change of rate of expansion (second order information). Luckily, the $\dot{\theta }$ signal grows in an exponential way and two temporally separated values of this variable before collision (but leaving time for a motor action) are sufficiently different (i.e., well above discrimination threshold) so that this difference can be reliably used with Equation (6).

**Figure 4:**
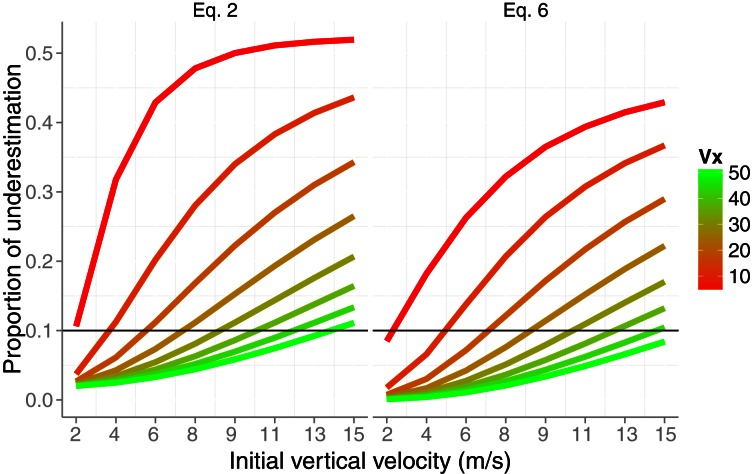
**Proportion of underestimation of the actual radial velocity *v*_*r*_ about 200 ms before contact as a function of the initial vertical velocity *v*_0*y*_.** Color codes different horizontal velocities. The underestimation is plot for Equations (2) and (6). For the sake of comparison, a reference horizontal line is added at the level of 10% of underestimation.

The dashed lines in Figure 3 show the estimation of the radial velocity that is obtained from Equation (6). In order to estimate *v*_*r*_ we slid Equation (6) along all the trajectory with different values of Δ*t*. We scrutinized Δ*t* from 0.016 to 0.320 s with steps of 0.016 s. For each value of Δ*t* we computed the squared difference between the actual radial velocity for the 64 trajectories (eight *v*_*x*_ × eight *v*_*v*_) and the estimate. The minimum squared error was obtained with Δ*t* = 0.096 s. This is the value used in the simulations shown in Figure 3. Although both methods underestimate the radial velocity, the procedure based on Equation (6) performs better. For the sake of comparison, Figure 4 shows the proportion of underestimation of *v*_*r*_ for the two methods. This proportion is computed about 0.2 s before contact. The estimates from Equation (6) are, consequently, better than those obtained by using Equation (2). For example, the level of underestimation for the fastest horizontal velocity (50 m/s) is always below the 10% of underestimation. Despite this improvement, however, radial velocity can only be recovered accurately for relatively fast horizontal velocities *v*_*x*_. Interestingly the better performance points to an optimal time window (Δ*t*) around 100 ms which is consistent with the time window of 100–140 ms of the low-pass filter proposed as a first stage to detect acceleration from speed changes (Werkhoven et al., 1992). Note that Equation (6) involves detecting a change of rate of expansion that is a second order change.

So far, we have left aside the effects of noise when computing the predictions for *TTC* based on the *KS* model, including the estimation of *v*_*r*_. Next we explore the behavior of the model taking into account this noise.

### Simulation of the *KS* model

Figures 2, 3 do not take into account the error introduced by the sensory system in estimating optical variables, that is the predictions shown so far are noise-free. However, behavioral and neural responses to optical variables θ and $\dot{\theta }$ in the initial part of the trajectory are very noisy signals (Keil and López-Moliner, 2012). They fluctuate due to the discrete structure of the retinal array and its limited spatial resolution. Because the signal-to-noise ratio improves as collision approaches, we would expect a differential effect of noise along the trajectory. In order to test the robustness of the *KS* model to sensory noise, we simulated Equations (6) and (5) (with $\dot{\gamma }=0$) for the different trajectories after adding Gaussian noise to θ and $\dot{\theta }$. Note that we need $\dot{\theta }$ to compute *v*_*r*_ (Equation 6). Similarly as in Keil and López-Moliner (2012), we start with adding Gaussian noise ξ with mean zero and standard deviation one to both angular variables:

(7) $$\begin{array}{lll} \theta_{t} & = & \theta_{t}+a_{\theta }\cdot \xi \\ {\dot{\theta }}_{t} & = & {\dot{\theta }}_{t}+a_{\dot{\theta }}\cdot \xi \end{array}$$

We simulated 10,000 runs of Equation (7) for each trajectory and the value of *a* was chosen so that the Weber fraction of θ (Δθ/θ) and $\dot{\theta }\;(\Delta \dot{\theta }/\dot{\theta })$ decreased with time according to reported values in the literature. For example, $\dot{\theta }$ converged to a Weber fraction of 10% (Regan and Hamstra, 1993) after 150 ms (Wurfel et al., 2005). In our case we used the value of one standard deviation across the runs for Δθ and $\Delta \dot{\theta }$ to compute the corresponding Weber fractions. To our knowledge, discrimination thresholds of θ have only been reported in static conditions and are about 4% (e.g., McKee and Welch, 1992), so we added the noise consistently with this discrimination threshold. For each trial we then computed *v*_*r*_ from Equation (6) (with a value of Δ*t* = 0.09 that provided the best estimate of *v*_*r*_ in Figure 3) and using the noisy versions of the optical variables θ and $\dot{\theta }$ and size *s* (Weber fraction for size: 4%). We low-pass filtered the output of Equation (6) to smooth velocity by using a 1st order low-pass filter (moving average) as in Keil and López-Moliner (2012) and finally applied Equation (5), the *KS* model with $\dot{\gamma }=0$.

**Figure 5:**
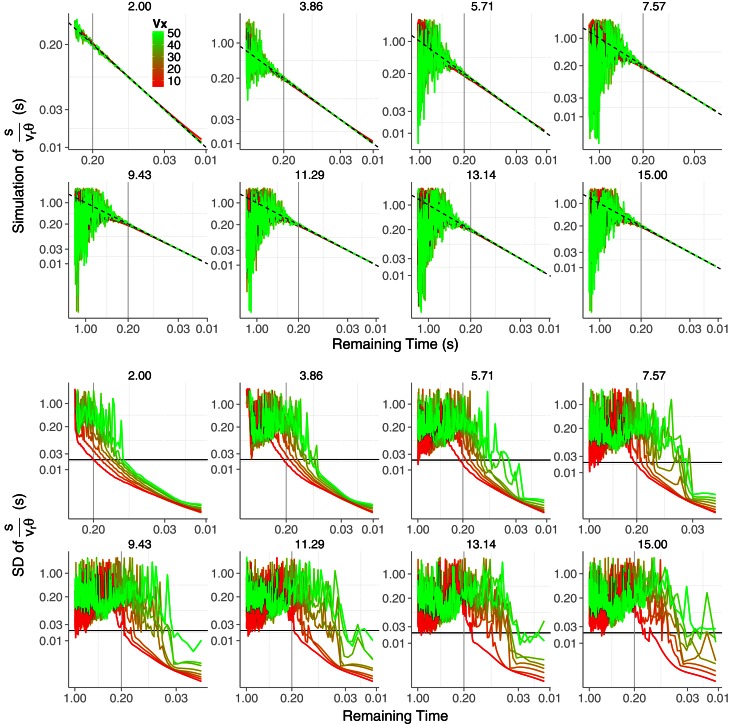
**Results of the simulation of the KS model Equation (5) with $\dot{\gamma }=0$.** (top panels) Predicted time error as a function of the remaining *TTC* in log–log coordinates. Different horizontal velocities *V*_*x*_ are color-coded and different *V*_0*y*_ are plotted in different panels. (bottom panels) Precision of the prediction (log–log coordinates). The horizontal line denotes an error of 20 ms. The gray vertical lines denotes a remaining time of 0.2 s. See text for simulation details. Axes have different scales from one panel to another to increase readability.

Figure 5 (upper panels) shows the simulated accuracy of the *TTC* for, at most, the last second of the trajectory. We show the data in a log–log space to increase the resolution at the moment of interest. As expected, the prediction is noisier as *v*_0*y*_ increases (one panel for each *v*_0*y*_). However, the accuracy is quite acceptable for all trajectories when it remains 0.2 s until contact (gray vertical line). Before this time, only for very low values of *v*_0*y*_ the prediction is reliable enough to be useful. As an indication of the precision, the standard deviation of the simulations are shown in the bottom panels of Figure 5. Again the expected variability only reaches useful values for interception (close to 20 ms denoted by the horizontal line) (Brenner and Smeets, 2009) at a useful time (e.g., between 200 and 100 ms before contact) for low values of *v*_0*y*_. In sum, these results render the *KS* model valid for catching a limited set of fly balls, namely, shallow trajectories in which the horizontal velocity is relatively higher than the initial vertical one.

**Figure 6:**
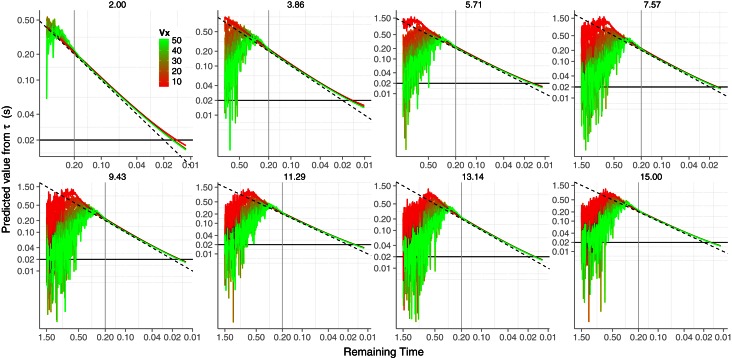
**Simulated signal of *TTC* if τ was used without any prior knowledge.** Color codes different horizontal velocities and each initial vertical velocity is plotted in a different panel. The horizontal black line denotes the 20 ms time error limit. The vertical gray line is placed at 0.2 s before contact, a useful time to have a good estimate in order to perform a catching action. Axes have different scales from one panel to another to increase readability.

One might wonder whether there is some chance for using τ directly in parabolic trajectories. Like the *KS* model, τ is a first-order approximation to *TTC* and is simply formulated as the ratio between visual angle and rate of expansion: $\theta /\dot{\theta }$. Recall that horizontal velocity *v*_*x*_ is constant along the trajectory and, therefore, when the initial vertical velocity is very small relative to the horizontal speed *v*_*x*_, the resultant trajectory is closer to a horizontal one. In addition, by using τ we circumvent the estimation of *v*_*r*_. To address this question we used the noisy versions of θ and $\dot{\theta }$ and computed the τ signal. No low-pass filter was applied to τ. In Figure 6 we plot (in log–log coordinates) the prediction error against the remaining *TTC*.

It can be seen that there is not much difference with the predicted error obtained with the previous simulations. Actually, τ can always accurately predict *TTC* about 200 ms before contact. For higher parabolae $\dot{\theta }$ is very low (e.g., closer to detection threshold values) for about half the flight time so that its measurement by the visual system is noisier resulting in a lower signal-to-noise ratio. This explains the large amount of noise, for example, when *v*_0*y*_ = 15 (last panel in Figure 6), especially when the horizontal speed is slow (red lines). The obtained precision (not shown) makes the viability of τ very unlikely.

## A model that includes size and gravity (*GS*) to estimate *TTC*

In the last section we have shown that previous models based on thresholding optical variables, be physical size known or not, have limited applicability to timing fly balls. Basically the limitation arises because these models rely on first-order visual information (e.g., recovering *v*_*r*_) and fail to provide an accurate and precise *TTC* when initial vertical velocity is high. Higher initial vertical velocities do indeed imply more visual contribution of the vertical component and consequently the use of $\dot{\theta }$ or *v*_*r*_ to obtain a first order approximation of *TTC* is seriously undermined. Despite the different kinds of visual information previously considered, it seems then that additional information would be needed to compensate for the acceleration/deceleration due to gravity. Accurate estimates of *TTC*, therefore, cannot be obtained from known size combined with optical variables or tau when the initial vertical velocity is much larger than the horizontal one (*v*_0*y*_ >> *v*_*x*_). Gravity's effects are ubiquitous. It is then logical to think that people can learn or adapt after long-life exposure to these effects. Some experiments have reported adaptation of motor responses to the effects of gravity (Lacquaniti and Maioli, 1987, 1989a,b) and more recently, a series of different studies have interpreted the ability to successfully intercept free falling objects when the fall was consistent with 1*g* as evidence for an internal model of gravity [e.g., Indovina et al., 2005; Senot et al., 2005, but see Baurès et al. (2007) for a critical appraisal and also the corresponding rebuttal by Zago et al. (2008)].

The behavioral evidence of good performance under gravity conditions has not, however, gone hand in hand with a feasible timing model that explains the level of performance. Lacquaniti and Maioli (1989b) proposed an algorithm for free-falling objects which relies on the online updating of the vertical speed:

(8) $$\begin{array}{l} TTC=\sqrt{\frac{2y_{0}}{g}}-\frac{\dot{y}}{g} \end{array}$$

In addition, the model needs input on the initial height, but this limitation is absent in other models (e.g., McIntyre et al., 2001; Zago et al., 2004) which only need a running estimate of the height *y* and its first temporal derivative *ẏ*. Apart from the problem of how *g* is represented, subjects would need to estimate the vertical speed in 3D space for these models to be useful. Correlates of this speed are certainly available from the optical or angular velocity $\dot{\gamma }$ in free falling balls if the distance between the line of fall and the observer's eyes can be estimated somehow (Zago et al., 2009).

However, the timing of parabolic trajectories which are probably the most frequent paths being affected by gravity remains to be solved. A previous attempt to obtain *TTC* in parabolic trajectories was made by Brouwer et al. (2006). In this study subjects did use $\dot{\gamma }$ to solve the outfielder problem task and the use of this variable is consistent with the model that it will be introduced later on. Unlike in the case of linear trajectories, the models considered so far for free-falling have made no explicit use of known size. We will next show that it is by considering both types of context-dependent variable (*g* and known size) that it is possible to formulate a relatively simple model for catching parabolic balls. In Appendix B we show that it is relatively straightforward to prove that an accurate estimation of *TTC* can be obtained using the following expression:

(9) $$TTC=\frac{2}{g}\frac{s\dot{\gamma }}{\theta \cos\gamma }$$

The *TTC* signal therefore would depend on two context-dependent variables (size *s* and gravity *g*), optical size θ, the elevation angle γ and its time derivative $\dot{\gamma }$. Note that expansion-like variables (i.e., $\dot{\theta }$ and *v*_*r*_) are not in Equation (9), which *a priori* makes this model more robust to noise because $\dot{\theta }$ is one of the noisiest optical measurements.

Next we will show that this model not only provides an accurate estimation of *TTC* but also high precision in this estimation. In addition, in Appendix B it is shown that only in very few parabolic cases (ones that are unlikely) is the model not viable.

### Simulation of the *GS* model

Using the same procedure as before we ran 10,000 simulations for each trajectory to test the noise suppression capacity of the *GS* model. We then added Gaussian noise to angular variables θ, γ and $\dot{\gamma }$:

(10) $$\begin{array}{lll} \theta_{t} & = & \theta_{t}+a_{\theta }\cdot \xi \\ \gamma_{t} & = & \gamma_{t}+a_{\gamma }\cdot \xi \\ {\dot{\gamma }}_{t} & = & {\dot{\gamma }}_{t}+a_{\dot{\gamma }}\cdot \xi \end{array}$$

**Figure 7:**
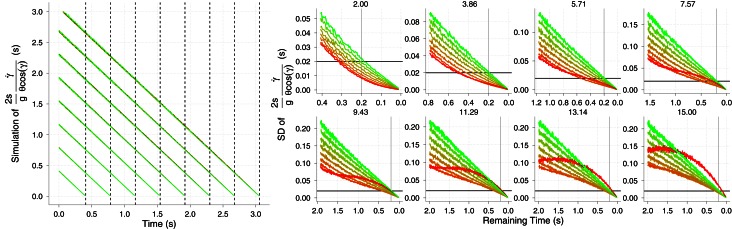
**Results of the simulation of model ***GS***. Left:** Simulated *TTC* accuracy of model *GS* as a function of elapsed time. Vertical dotted lines denote the *TTC* for the different *v*_0*y*_. **Right:** Simulated temporal SD (reciprocal of precision) of the model. As before, different initial vertical velocities are plotted in different panels and horizontal black lines and vertical gray lines denote 20 ms. of precision error and 0.2 s. before contact. See text for details on the computation of the temporal precision. Color codes different horizontal velocities *v*_*x*_ as in Figure 6. Axes have different scales from one panel to another to increase readability.

For θ we proceed in the same way as with the simulation of the *KS* model, and so we noisified physical size. For γ and $\dot{\gamma }$ we set *a* so that both converged to a Weber fraction of 5%. We did not add any noise to *g*. The results of the simulations are shown in Figure 7. Accuracy of the *GS* model in predicting *TTC* is very high from the very beginning of the trajectory (see Figure 7 left). The differences between different horizontal velocities *v*_*x*_ (color coded) and the level of noise are unnoticeable. Therefore, the *GS* model is very robust to noise along all the trajectory. Note that, unlike θ, the signal-to-noise ratio is very high at the beginning and decreases with time, so that the noisiest measurements of $\dot{\gamma }$ do not occur at the same time as those for θ. More interestingly, the precision of the *TTC* estimation is quite high (SD near 20 ms) about 200 ms before contact (Figure 7 right). One second before contact (for longer *TTC*) the level of precision is around 100 ms, which could be enough to enable start anticipatory movements that can be further refined. These figures place the *GS* model in a good position to be further explored in the context of interceptive timing. The reported accuracy and precision values were obtained with simulating trajectories that are likely to happen in ball games (e.g., tennis, cricket) and would enable both prospective and on-line strategies. We will revisit this point in the discussion. However, we have so far neglected air drag in the *GS* model and it might well be an important matter. We touch on this issue in the next section.

### Effect of air drag on the *GS* model

If we omit air drag the ball acceleration for the horizontal and vertical components are, respectively *a*_*x*_ = 0 and *a*_*y*_ = −*g*, so that the horizontal velocity *v*_*x*_ remains constant while the vertical velocity changes due to gravity: first decelerates and then accelerates again. If we consider air drag, then the air drag force *f* is approximately proportional to the square of the ball's tangential speed (*v* in Figure 1) (Timmerman and van der Weele, 1999):

$$f\approx Dv^{2}$$

where *D* is a constant that can be further decomposed in several parts:

$$D=\frac{1}{2}\rho \cdot C\cdot A$$

with *A* being the sectional area of the ball (*A* = π · radius^2^); ρ is the density of the air and *C* is a dimensionless constant called drag coefficient and it depends on the shape of the object. For spherical objects like tennis balls *C* is about 0.4–0.5 at sea level (Timmerman and van der Weele, 1999). The acceleration components including air drag then become:

(11) $$\begin{array}{l} a_{x}=-\frac{D}{m}v\cdot v_{x}\\ a_{y}=-g-\frac{D}{m}v\cdot v_{y} \end{array}$$

where *m* is the mass of the ball. Once we have the starting velocities (*v*_*x*_ and *v*_*y*_) and positions (*x* and *y*) we can proceed with updating these variables taking into account air drag:

(12) $$v_{x}+\Delta v_{x}=v_{x}+a_{x}\Delta t;\;v_{y}+\Delta v_{y}=v_{y}+a_{y}\Delta t$$

(13) $$\begin{array}{l} x+\Delta x=x+v_{x}\Delta t+\frac{1}{2}a_{x}{\left(\Delta t\right)}^{2};\\ y+\Delta y=y+v_{y}\Delta t+\frac{1}{2}a_{y}{\left(\Delta t\right)}^{2} \end{array}$$

**Figure 8:**
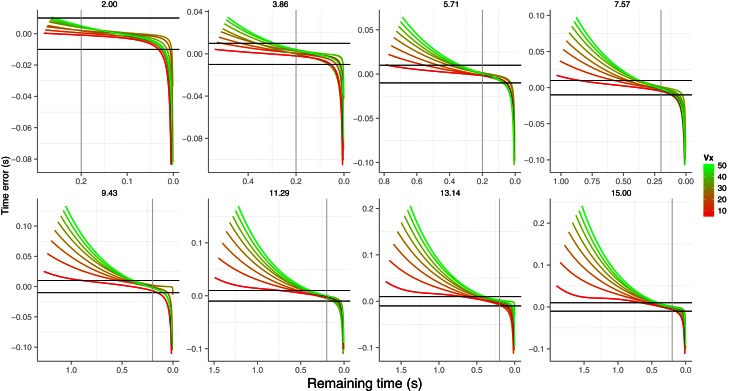
**Predicted estimation error of the mode *GS* considering air drag for the last 70% of the trajectory.** Different initial vertical velocities are plot in different panels. The two solid horizontal lines denote a time window of 20 ms and the gray horizontal lines signal a remaining arrival time of 0.2 s. Color codes different *v*_*x*_. Axes have different scales from one panel to another to increase readability.

Therefore with a sufficiently small value of Δ*t* we can numerically simulate the parabolic trajectories considering air drag and update the optical variables of Equation (9) according to the new kinematics. We did so for the 64 trajectories and could compute the temporal error resulting from applying the *GS* model. We simulated the situation of a tennis ball (mass of 0.057 Kg) with ρ = 1.2 and *C* = 0.5 which are proper values for a sea level. Figure 8 shows the temporal error, that is the difference between the prediction from the *GS* model and the actual arrival time. The error is shown for the last 70% of the trajectory. As can be noted, the model does not predict the exact remaining time any longer (the error would be zero and constant along the trajectory). However, the error remains very small and within an acceptable range during the most relevant parts of the trajectory. The two horizontal lines denote the range of a time window of 20 ms. For the smaller initial vertical velocity (*v*_0*y*_ = 2 m/s), for example, the predicted error is very small up to the very last frames of the trajectory. In any case, the error is always within acceptable limits about 0.2 s before contact rendering the *GS* model valid in order to account for real-life performance. Interestingly, we did not change the value of *g*, therefore how the brain represents (e.g., integrated versus fragmented) the forces that affect vertical velocity (i.e., through gravity and air drag) would be an interesting problem for future research.

## Discussion

We have here advanced two possible models that are capable of estimating *TTC* for fly balls using monocular variables only. Catching a ball in real-life not only implies getting the timing right, but also to be at the correct position. One important caveat to bear in mind, therefore, is that the reported predictions, both theoretically-derived and simulated, assume an optic flow consistent with a direct approach, or, put another way, the fly ball was in a direct collision path with the observer's eyes. Despite this being, of course, a simplification which has often been made in the *TTC* literature, it allowed for the first time to characterize useful models for obtaining *TTC* estimates with fly balls. In this sense, solving the outfielder problem [see Fink et al. (2009) for a recent critical test] could be regarded as a complementary previous step where the catcher runs to the correct place (spatial problem) to be able to efficiently use optical flow to solve the timing side of catching.

The two models that we have introduced are *a priori* reliable candidates to deal with this timing. The Known-Size (*KS*) model extends previous solutions that have been derived elsewhere (López-Moliner et al., 2007b; López-Moliner and Keil, 2012) to estimate *TTC* for linear trajectories. Nevertheless, the model has limited applicability to parabolic trajectories and, except for shallower parabolae, its use is not feasible as a general model for catching in ball games, for example. Because this model directly relies on θ and indirectly on $\dot{\theta }$ to estimate *v*_*r*_, the signal-to-noise ratio is very low in the early part of the trajectory. The poor capacity of noise suppression during the early approach has also been reported for τ in Keil and López-Moliner (2012). This aspect makes all these models that are based on first-order estimation only useful in the late part of the ball flight. Given that enough time is available to perform a catching actions (e.g., 200 ms), this model therefore cannot be rule out and may be well applied when horizontal velocity is higher than the vertical one (e.g., *v*_0*y*_ < 5 in our simulations). The *KS* model can, however, gain further support in the context of control models of interception. Predictive versus on-line control strategies are the two contrasting views in the literature. The former type of control can sometimes be arguably associated with using some internal model [e.g., visual representation of gravity (Indovina et al., 2005)] that can make a temporal prediction and thereby circumvent neural delays. *A priori*, therefore, little role is expected for the *KS* model from this perspective, as it only signals reliably *TTC* in the last part of the fly. However, leaving enough room to respond, the system could rely on this first-order model until the very last possible moment Lacquaniti and Maioli (1989b). The very low predictive potential of the *KS* model makes it difficult to be reliable to initiate pre-programmed movements at critical times (Tresilian and Houseman, 2005) because subjects could hardly use the model to estimate the moment of interest in advance.

**Figure 9:**
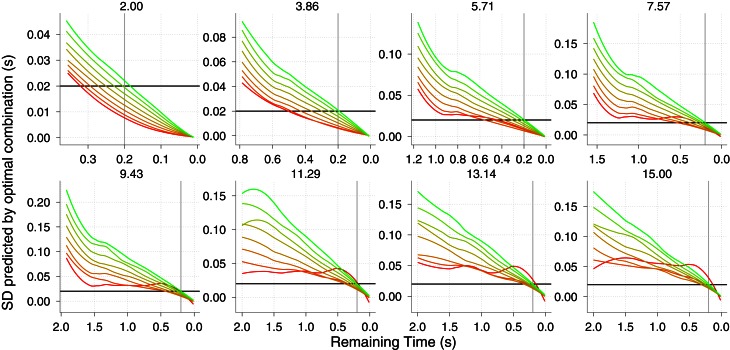
**Predicted SD (variable error) if the two models *KS* and *GS* were integrated in an optimal fashion.** The resulting variance is time-wise computed as follows: (σ^2^_*ks*_ × σ^2^_*gs*_)/(σ^2^_*ks*_ + σ^2^_*gs*_) Different panels show different *v*_0*y*_. A local Gaussian smoothing has been applied to the predictions. The horizontal black line and the vertical gray line denote a precision of 20 ms and 0.2 s before contact, respectively. Color codes different *v*_*x*_ as in Figures 6, 7. Axes have different scales from one panel to another to increase readability.

Unlike the *KS* model, the *GS* does signal correct *TTC* with relatively high precision well in advance (σ ≈ 100 ms 1 s before contact). So one of the main benefits of the *GS* model over the *KS* one is the predictive potential. The use of the *GS* model could then be tested by showing incremental parts of the parabolic trajectory and see whether the response precision in initiating the action evolves as predicted (see Figure 7 right). *A priori* it would be difficult to favor either model by looking at predicted precision or accuracy when only late information is shown. However, we do not consider different sources of information for *TTC* as being mutually exclusive, but rather likely to be integrated. In this regard, optimal combination provides a nice framework to test whether subjects would reduce their variability following maximum likelihood when late parts of the trajectory are shown. If so the resulting variability will be less than the minimum variability predicted from using the *GS* model. Figure 9 shows the predicted precision if both models were combined optimally. Note that there is a clear benefit (compared with Figure 7 right) specially for high *v*_0*y*_ and slow *v*_*x*_ (red color) where the precision 2 s before contact is increased by 50 ms.

**Figure 10:**
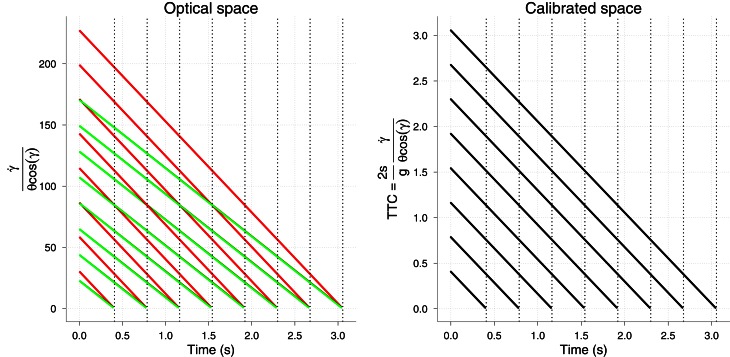
**Left:** Output of the *GS* model but considering optical variables only. The two colors denote two different physical sizes (66 and 88 cm of diameter). **Right:** Final output of the model *GS* in which the two sizes are normalized and the correct *TTC* is signaled for both sizes. Vertical lines denote the *TTC* for each *v*_0*y*_.

Finally, there is the issue of how prior knowledge, which is required in the *GS* model, is implemented. As in López-Moliner et al. (2007b) or Zago et al. (2008) neither are size or gravity, respectively considered as being higher-level cognitive representations, but rather a place-holder for variables in optical space. Let us illustrate this idea by using the *GS* model. Figure 10 (left) shows the *optical* output of the model for two different sizes. Note that the resulting optical output decreases linearly with time for both sizes, but the actual values are meaningless as to *TTC*. On Earth these values are experienced by an observer sensory system and associated with a physical ball size in a given context through learning. Importantly, note that different *v*_*x*_ collapse so that the time course of the optical output is independent of different horizontal velocities. The normalization across different *v*_*x*_ is achieved through the term cos γ. Both a fixed physical size and constant *g* calibrate or normalize the optical values so that they are properly interpreted by the system to signal *TTC*. As a consequence, the use of prior values of size or gravity by the *GS* model could be tested in a similar fashion as in López-Moliner et al. (2007b) and López-Moliner and Keil (2012). Physical size helps the system to interpret the optic flow and obtain a useful threshold as an indicator to start the action. In the *GS* model, then, physical size (s) would provide the metrics for interpreting (or calibrating) θ. Suppose one person has learnt to start acting at a corresponding threshold (θ_*t*_) when you throw her a tennis ball. Later she is suddenly presented with a football ball whose projected retinal image matches the smaller geometry of the tennis ball. The prior information triggered by familiarity cues (denoting a football ball) would set the new threshold to a larger value so that the remaining time at action onset is invariant within the task. Since the image will grow at a lower rate than expected the new threshold is reached at a later time than it would the real image of a football ball. This leads to the overestimation of the *TTC* in this sort of situation and this very same pattern has been reported for this type of manipulation in Hosking and Crassini (2010) when displaying parabolic trajectories. Although the optical geometry that was used in this study was not exactly the same as in our simulations, our proposal would then be consistent with their results.

In this calibration process, multisensory feedback about our success in catching might play a relevant role. Knowledge of physical size is necessary to modulate the closing of the hand when catching a ball. The knowledge of size that we obtain from the haptic feedback in catching is much more robust than that obtained from the visual system. Just as the haptic system has been proposed to educate the visual system as to size perception (Gori et al., 2008, 2011), a similar calibration process might be happening for *TTC* relevant optical variables.

### Conflict of interest statement

The authors declare that the research was conducted in the absence of any commercial or financial relationships that could be construed as a potential conflict of interest.

## Appendix A

### Mathematical derivation of the model *KS*

In this appendix we derive the equation for the model *KS* which extends prior models of TTC for horizontal movements to parabolic ones. In order to do so, it is necessary to obtain *v*_*x*_ in terms of visual parameters that can be estimated by the catcher. Therefore we will express *v*_*x*_ in terms of *v*_*r*_ and *v*_*t*_. In order to do so, we first define the components of a generic parabolic movement as illustrated in Figure 1:

- (*x*(*t*), *y*(*t*)) = (*x*, *y*) is the position of the object at time *t*.
- *y*_*m*_ is the maximum height of the object along the trajectory.
- *x*_*m*_ is the corresponding horizontal value (abscise) when the object reaches the maximum height *y*_*m*_.
- *v*_0*y*_ is the initial vertical component of velocity.
- *v*_*y*_ is the vertical component of velocity.
- *v*_*x*_ is the horizontal component of velocity.
- *v* is the velocity of the ball which is tangent to the parabola.
- *v*_*r*_ is the radial component of velocity.
- *v*_*t*_ is the tangential component of velocity with respect to the point of observation.
- *d* is the distance between the object and the catcher's eyes.

Note that we set time *t* = 0 when the ball is at the origin of coordinates [point (0,0)]. We denote by *t*_*m*_ = *v*_0*y*_/*g* the time the object takes to rise from *y* = 0 to *y* = *y*_*m*_ or to descend from *y* = *y*_*m*_ to *y* = 0. With these definitions we can easily derive the following equations for the parabolic movement:

$$\begin{array}{l} \;x(t_{m})=x_{m}=\frac{v_{x}v_{0y}}{g}\\ \;y(t_{m})=y_{m}=\frac{v_{0y}^{2}}{2g}\\ x(2t_{m})=2x_{m}=X_{m}=\frac{2v_{x}v_{0y}}{g}\\ \;y(2t_{m})=0\\ \;x(t)=v_{x}t\\ \;y(t)=v_{0y}t-\frac{gt^{2}}{2}\\ \;v_{y}=v_{0y}-gt \end{array}$$

In addition, by applying trigonometry to isolate *v*_*r*_ and *v*_*t*_ in Figure 1 we have:

$$v_{r}=\frac{(X_{m}-x)v_{x}-yv_{y}}{d}=\frac{(X_{m}-x)v_{x}-yv_{y}}{\sqrt{{\left(X_{m}-x\right)}^{2}+y^{2}}}$$

and

$$v_{t}=\frac{(X_{m}-x)v_{y}+yv_{x}}{d}=\frac{(X_{m}-x)v_{y}+yv_{x}}{\sqrt{{\left(X_{m}-x\right)}^{2}+y^{2}}}$$

Next, we need to introduce the size of the object *s*, visual angle θ, and rate of expansion $\dot{\theta }$.

Let us consider that the ball has diameter *s*, and is at distance *d* of the catcher (see Figure 1). It verifies that tan(θ) = *s*/*d*. However, if the object is spheric, it can be proved that the relation between θ, *s* and *d* is (see inset of Figure 1):

(A.1) $$\sin(\theta /2)=\frac{s/2}{d}$$

For the sake of simplicity, however, and given that *s*/*d* is small, the following equality is often used without a significant loss of precision:

(A.2) $$d\approx \frac{s}{\theta }$$

Next, we are going to extent two models to estimate *TTC* for horizontal movement. First, if a threshold of θ is used, the following expression can be used to compute *TTC* (Sun and Frost, 1998; López-Moliner and Bonnet, 2002):

(A.3) $$TTC=\frac{s}{v_{x}\theta }$$

Equation (A.3) for horizontal movements can be easily derived by combining Equation (A.2) and *T*_*c*_ = (*X*_*m*_ − *x*)/*v*_*x*_ = *d*/*v*_*x*_

Let us generalize Equation (A.3) to parabolic movements. According to Figure 1 we have

$$v_{x}=v_{r}\cos\gamma +v_{t}\sin\gamma \approx v_{r}\cos\gamma +\dot{\gamma }\frac{s}{\theta }\sin\gamma$$

where we have used that $v_{t}=\dot{\gamma }d\approx \dot{\gamma }s/\theta$. Therefore:

(A.4) $$\begin{array}{l} TTC=\frac{X_{m}-x}{v_{x}}=\frac{s\cos(\gamma )/\theta }{v_{r}\cos\gamma +\dot{\gamma }s\sin(\gamma )/\theta }=\frac{s}{v_{r}\theta +s\dot{\gamma }\tan\gamma } \end{array}$$

Observe that when tan γ = 0 Equation (A.4) becomes Equation (A.3). Let us see that Equation (A.4) also contains the estimation of *TTC* based on a $\dot{\theta }$ threshold. If we derive Equation (A.2) with respect to time we have $-v_{r}=-s\dot{\theta }/\theta^{2}$. In addition, for any horizontal movement we have that *v*_*r*_ = *v*_*x*_ and *v*_*r*_/*d* = 1/*T*_*c*_.

If we combine these expressions with Equation (A.4) we then have for horizontal movements:

(A.5) $$T_{c}=\frac{s}{v_{r}\dot{\theta }T_{c}}$$

The positive solution of Equation (A.5) is:

(A.6) $$TTC=\sqrt{\frac{s}{v_{r}\dot{\theta }}}$$

which turns out to be the model that specifies *TTC* for linear trajectories based on a $\dot{\theta }$ threshold, given that size and velocity are known (López-Moliner et al., 2007b).

## Appendix B

### Mathematical derivation of the model including physical size and gravity *GS*

We here derive the model represented in Equation (9). According to Figure 1 we can express the elevation angle γ as follows:

(B.1) $$\begin{array}{lll} \gamma & = & \arctan\left(\frac{v_{0y}t-\frac{gt^{2}}{2}}{X_{m}-v_{x}t}\right)=\arctan\left(\frac{g}{2v_{x}}\frac{t^{2}-\frac{2v_{0y}}{g}t}{t-\frac{X_{m}}{v_{x}}}\right)\\ & = & \arctan\left(\frac{g}{2v_{x}}t\right) \end{array}$$

where we have used that

$$\frac{2v_{0y}}{g}=\frac{X_{m}}{v_{x}}$$

and $t\neq \frac{X_{m}}{v_{x}}$. That is, the ball is not at the catcher place: (*X*_*m*_, 0).

Then, using the derivative of the arctan function:

$$\frac{d\arctan at}{dt}=\frac{a}{1+{\left(at\right)}^{2}}$$

we obtain the following expression for $\dot{\gamma }$:

(B.2) $$\dot{\gamma }=g\frac{2v_{x}}{{\left(2v_{x}\right)}^{2}+{\left(gt\right)}^{2}}=g\frac{2v_{x}}{{\left(2v_{x}\right)}^{2}+{\left(v_{0y}-v_{y}\right)}^{2}}$$

Moreover, isolating *gt* from Equation (B.1) we have:

(B.3) $$gt=2v_{x}\tan\gamma$$

When *gt* of Equation (B.3) is substituted into Equation (B.2), we have:

$$\dot{\gamma }=\frac{g}{2v_{x}}\frac{1}{1+\tan^{2}\gamma }=\frac{g}{2v_{x}}\cos^{2}\gamma$$

Then,

(B.4) $$v_{x}=\frac{g}{2\dot{\gamma }}\cos^{2}\gamma$$

In addition, using Equation (A.1) we have:

(B.5) $$X_{m}-x=\sqrt{{\left(X_{m}-x\right)}^{2}+y^{2}}\cos\gamma \approx \frac{s}{\theta }\cos\gamma$$

Then, combining Equations (B.4) and (B.5) we have that the time to contact *TTC* is:

(B.6) $$TTC=\frac{X_{m}-x}{v_{x}}=\frac{s}{\theta }\cos\gamma \frac{2\dot{\gamma }}{g\cos^{2}\gamma }=\frac{2}{g}\frac{s\dot{\gamma }}{\theta \cos\gamma }\;(GS)$$

which denotes the model *GS*.

#### Limitations of using the *GS* model due to the precision of estimating $\dot{\gamma }$

Since the discrimination threshold of $\dot{\gamma }$ is about 5% between 0.03 and 1.2 rad/s (McKee, 1981; Regan, 1997), there could be cases in which the value of $\dot{\gamma }$ falls out of this window and could undermine the use of the model. Here we derive the cases in which the precision of $\dot{\gamma }$ is not warranted. Let us consider the interval at which $\dot{\gamma }$ can be estimated with a 5% error is between 0.03 and 1.2 rad s^−1^, which we denote by $\left(0.03,\;1.2)=({\dot{\gamma }}_{\text{min}},\;{\dot{\gamma }}_{\text{max}}\right)$.

Let us see now when it is possible to estimate $\dot{\gamma }$ with this precision. According to Equation (B.2), when

$${\dot{\gamma }}_{\text{min}}<g\frac{2v_{x}}{{\left(2v_{x}\right)}^{2}+{\left(gt\right)}^{2}}=g\frac{2v_{x}}{4v_{x}^{2}+{\left(v_{0y}-v_{y}\right)}^{2}}<{\dot{\gamma }}_{\text{max}}$$

we can then estimate *TTC* according to Equation (B.6) with a high precision.

The time a ball is above its initial heigh is given by:

(B.7) $$\begin{array}{l} t=\frac{2v_{0y}}{g} \end{array}$$

Because we assume that the maximum time is the time denoted by Equation (B.7), when the object is moving *t* must then satisfy

(B.8) $$0<t<\frac{2v_{0y}}{g}$$

However, when we want to estimate *TTC* according to Equation (B.6) without being affected by a loss of precision in estimating $\dot{\gamma }$, after rearranging the inequalities shown just before B.7, *t* should also satisfy

(B.9) $$\frac{1}{g}\sqrt{\frac{2g}{{\dot{\gamma }}_{\text{max}}}v_{x}-4v_{x}^{2}}<t<\frac{1}{g}\sqrt{\frac{2g}{{\dot{\gamma }}_{\text{min}}}v_{x}-4v_{x}^{2}}$$

as well.

That is,

$$\frac{2}{9.81}\sqrt{4.0875v_{x}-v_{x}^{2}}=t_{1}<t<t_{2}=\frac{2}{9.81}\sqrt{163.5v_{x}-v_{x}^{2}}$$

There is an optimal time interval in which $\dot{\gamma }$ will be estimated with 5% precision. Therefore, these values of *t* such that also satisfy inequalities (B.8) and inequalities (B.9) are:

- Case 1: 0 < *v*_*x*_ < 4.0875 m/s
  In this case, *t*_1_ < *t* < min{*t*_2_, 2*v*_0*y*_/*g*}
- Case 2: 4.0875 m/s < *v*_*x*_ ≤ 163.5 m/s
  In this case, *t* < min{*t*_2_, 2*v*_0*y*_/*g*}

Case 1 represents the flights where the initial value of $\dot{\gamma }$ will be larger than 1.2 deg/s (because the horizontal velocity *v*_*x*_ is smaller than 4.08 m/s) and will decrease and eventually be within the optimal interval at time *t* so that *t* > *t*_1_. However, two cases are possible in which *t* < *t*_1_:

- a1: The initial *TTC* will be large enough (about 600 ms) and it would be possible to wait until time *t* to estimate $\dot{\gamma }$ accurately. This will happen when *v*_0*y*_ > 2 and the use of the *GS* model would become possible.

- a2: The value of *v*_0*y*_ is very small and so is the initial *TTC* (400 ms or less). Then it is not possible to wait until the time *t* at which $\dot{\gamma }$ is below 1.2. This will happen when *v*_0*y*_ < 3 which combined with *v*_*x*_ < 4.08 results in a very short distance parabolae (about 30–40 cm).

Case 2 is violated when *v*_*x*_ > 163.5 m/s or very near to this speed. The rate of the elevation angle $\dot{\gamma }$ will then always be below 0.03 deg/s, that is *t*_2_ < *t*. This situation (*v*_*x*_ ≈ 163.5 m/s, about 590 km/h) is rather unusual and indeed impossible in ball games.
